# Revisiting area risk classification of visceral leishmaniasis in Brazil

**DOI:** 10.1186/s12879-018-3564-0

**Published:** 2019-01-03

**Authors:** Gustavo Machado, Julio Alvarez, Haakon Christopher Bakka, Andres Perez, Lucas Edel Donato, Francisco Edilson de Ferreira Lima Júnior, Renato Vieira Alves, Victor Javier Del Rio Vilas

**Affiliations:** 1 Department of Population Health and Pathobiology, College of Veterinary Medicine, North Carolina State University, 1060 William Moore Drive, Raleigh, NC 27607 USA; 2 VISAVET Health Surveillance Center, Universidad Complutense, Avda Puerta de Hierro S/N, 28040 Madrid, Spain; 3 Departamento de Sanidad Animal, Facultad de Veterinaria, Universidad Complutense, Avda Puerta de Hierro S/N, 28040 Madrid, Spain; 4 CEMSE Division, King Abdullah University of Science and Technology, Trondheim, Saudi Arabia; 5 Department of Veterinary Population Medicine, College of Veterinary Medicine, University of Minnesota, St Paul, MN 55108 USA; 6 Secretaria de Vigilância em Saúde, Ministério da Saúde (SVS-MH), Brasília, Brazil; 7 School of Veterinary Medicine, University of Surrey, Guildford, Surrey GU2 7A, UK

**Keywords:** Visceral leishmaniasis, Brazil, Disease mapping, Bayesian, Risk classification

## Abstract

**Background:**

Visceral leishmaniasis (VL) is a neglected tropical disease of public health relevance in Brazil. To prioritize disease control measures, the Secretaria de Vigilância em Saúde of Brazil’s Ministry of Health (SVS/MH) uses retrospective human case counts from VL surveillance data to inform a municipality-based risk classification. In this study, we compared the underlying VL risk, using a spatiotemporal explicit Bayesian hierarchical model (BHM), with the risk classification currently in use by the Brazil’s Ministry of Health. We aim to assess how well the current risk classes capture the underlying VL risk as modelled by the BHM.

**Methods:**

Annual counts of human VL cases and the population at risk for all Brazil’s 5564 municipalities between 2004 and 2014 were used to fit a relative risk BHM. We then computed the predicted counts and exceedence risk for each municipality and classified them into four categories to allow comparison with the four risk categories by the SVS/MH.

**Results:**

Municipalities identified as high-risk by the model partially agreed with the current risk classification by the SVS/MH. Our results suggest that counts of VL cases may suffice as general indicators of the underlying risk, but can underestimate risks, especially in areas with intense transmission.

**Conclusion:**

According to our BHM the SVS/MH risk classification underestimated the risk in several municipalities with moderate to intense VL transmission. Newly identified high-risk areas should be further evaluated to identify potential risk factors and assess the needs for additional surveillance and mitigation efforts.

**Electronic supplementary material:**

The online version of this article (10.1186/s12879-018-3564-0) contains supplementary material, which is available to authorized users.

## Background

Visceral leishmaniasis (VL) in the Americas is a vector-borne neglected zoonosis caused by the intracellular protozoan *Leishmania infantum* [1, 2]. If left untreated, VL is fatal in more than 90% of cases, within two years of the onset of the disease [3].

Every year approximately 200,000–400,000 new cases of VL are registered worldwide [4]. In 2015, 88.8% of VL cases were reported from six countries: Brazil, Ethiopia, India, Somalia, South Sudan and Sudan [4], Brazil was ranked second, reporting 3289 new cases, 14% of the total reported worldwide, surpassed only by India [5]. In the Americas, Brazil represents 95% of total occurrences [6].

In Latin America transmission is mediated by the vector *Lutzomyia longipalpis* and *Lutzomyia cruzi* [7–9], a synanthropic sandfly with a wide geographic distribution in Brazil [10], and the domestic dogs as its the main animal reservoir in urban and rural areas. Control measures applied against the vector and the reservoir have shown limited success [11].

The Secretaria de Vigilância em Saúde of Brazil’s Ministry of Health (SVS/MH) is responsible for the planning, implementation and evaluation of VL surveillance in Brazil. VL surveillance data is used by the SVS/MH for the classification of municipalities in four VL risk categories. This risk classification is the main pillar for the management of the VL control in the country, and is currently based on the average number of reported cases per municipality in periods of 3-years, without considering human population at risk. Such simple classification and ranking approach does not account for uncertainties around the average number of cases and variability around risk metrics, and may be unable to fully recognize and address spatial and spatiotemporal dependencies in the data [12].

In this study, we evaluate the spatiotemporal pattern of VL risk in Brazil and generate alternative risk categories to compare with the current SVS/MH risk-classification. We aim to provide additional insights in the epidemiology of VL in Brazil, and inform how accurately the current risk categories reflect the underlying VL risk at the municipality level.

## Methods

### Data source and collection

The study area comprised all 5564 municipalities in Brazil as listed by the Instituto Brasileiro de Geografia e Estatística (IBGE) database (IBGE general information http://www.ibge.gov.br/english/). Municipality-specific annual counts of VL cases for the period 2004–2014, and the official risk classification status for the period 2008–2014 were provided by the SVS/MH.

### Data analysis

In order to account for the population at risk, we computed the municipality-specific standardized incidence ratios (SIR), ${SIR}_{it}=\frac{y_{it}}{e_{it}}$, where, for municipality *i* and year *t, y*_*it*_ is the count of VL cases and *e*_*it*_ the expected number of cases calculated by multiplying the population in municipality *i* for the *t* year (based on 2010 national census data) by the incidence of VL in the country.

At the first level of the BHM, the observed number of human VL cases in municipality *i* and year *t* (*y*_it_) was assumed to follow a Poisson distribution *y*_it_ ~ Poisson (*e*_it_, *θ*_it_), where *e*_it_ is defined above and *θ*_it_ is the unknown municipality-specific annual relative risk.

The log of *θ*_it_ was then decomposed additively into spatial and temporal effects and a space-time interaction term, so that

$$ Log\ \left({\theta}_{it}\right)=\alpha +{\upsilon}_i+{\nu}_i+{\gamma}_t+{\delta}_{it} $$

where *α* is the intercept, representing the population average risk, *υ*_*i*_ and *ν*_*i*_ describe respectively the spatially structured and unstructured variation in VL risk, *γ*_*t*_ represents the structured temporal effect, and *δ*_*it*_ is a space-time interaction term where given by the Kronecker product *γ*_*t*⨂_ *υ*_*i*_. Given the large number of municipalities with zero case counts we explored other parameterizations, specifically a zero inflated Poisson likelihood. We computed the Deviance Information Criterion (DIC) to compare the fit of our models [13].

A non-informative normal distribution with mean 0 and variance${\sigma}_{\nu}^2$ was used as prior distribution for the spatially unstructured random effect *ν*_*i*_, while the spatially structured effect *υ*_*i*_ was assigned a conditional autoregressive structure as previously described [14]. Briefly, *υ*_*i*_ was assumed to follow a normal distribution with mean conditional to the neighboring municipalities *υ*_*j*_, where neighborhood is defined in terms of geographical adjacency, and variance ${\sigma}_{\upsilon}^2$ dependent on the number of neighboring municipalities *n*_*i*_,

$$ {\upsilon}_i\mid \upsilon, j\  neighbor\ of\ i\sim N\ \left(\frac{1}{n_i}\ \gamma {\sum}_{j=1}^{n_i}{\upsilon}_j,\frac{\sigma_{\upsilon}^2}{n_i}\right) $$

Finally, *γ*_*t*_ was assigned a random walk type 1 (RW1) ${\gamma}_t\sim N\left({\gamma}_{t-1},{\sigma}_{\gamma}^{-1}\right)$. Exponential priors (3, 0.01) were assigned to all the standard deviations of the random effects [15]. In addition, we investigated also the sensitivity of our results to other less informative priors with larger ranges. Model posterior parameters were estimated using Integrated Nested Laplace Approximation (INLA), and fitted using the R-INLA package [16] conducted in R [17]. Results were visualized using ArcGIS 10.4 (ESRI ArcMap, 2016).

### Risk classification

The actual MH risk classification is based upon the most recent three-year moving average of the number of VL human cases registered in each municipality. This classification is updated every June. Municipalities are classified as no transmission (class 0, no cases reported), sporadic transmission (class 1, moving average < 2.4), moderate transmission (class 2, moving average in the interval [2.4–4.4), and intense transmission (class 3, moving average ≥ 4.4 cases) [18, 19].

In order to compare the current risk classification SVS/MH with the results of the BHM, we computed the posterior estimates of the ‘exceedence’ probability of risk *Prob* (*θ*_*it*_ > 1) ∣ *y* [20–22] further categorized into 4 categories (0, 1, 2, 3) if *Prob*(*θ*_*it*_ > 1) assumed < 0.5, 0.5–0.75, 0.75–0.95 and >  0.95, respectively. Exceedence categories were compared with the four SVS/MH risk classes via the weighted Kappa correlation test. Finally, the correspondent three-year moving average of the annual number of cases per municipality predicted by the model BHM ($\hat{y_{it}}$) was used to create a third risk classification in which municipalities were classified as no transmission (class 0, no cases predicted); sporadic transmission (class 1, $\hat{y_{it}}$ predicted moving average < 2.4); moderate transmission (class 2, $\hat{y_{it}}$ predicted moving average in the interval 2.4–4.4) and intense transmission (class 3, $\hat{y_{it}}$ predicted moving average ≥ 4.4) to compare with the SVS/MH classification based on observed cases (*y*_it_). The agreement between this classification and that of the SVS/MH was also compared via the weighted Kappa correlation test.

## Results

### Descriptive results

**Fig. 1:**
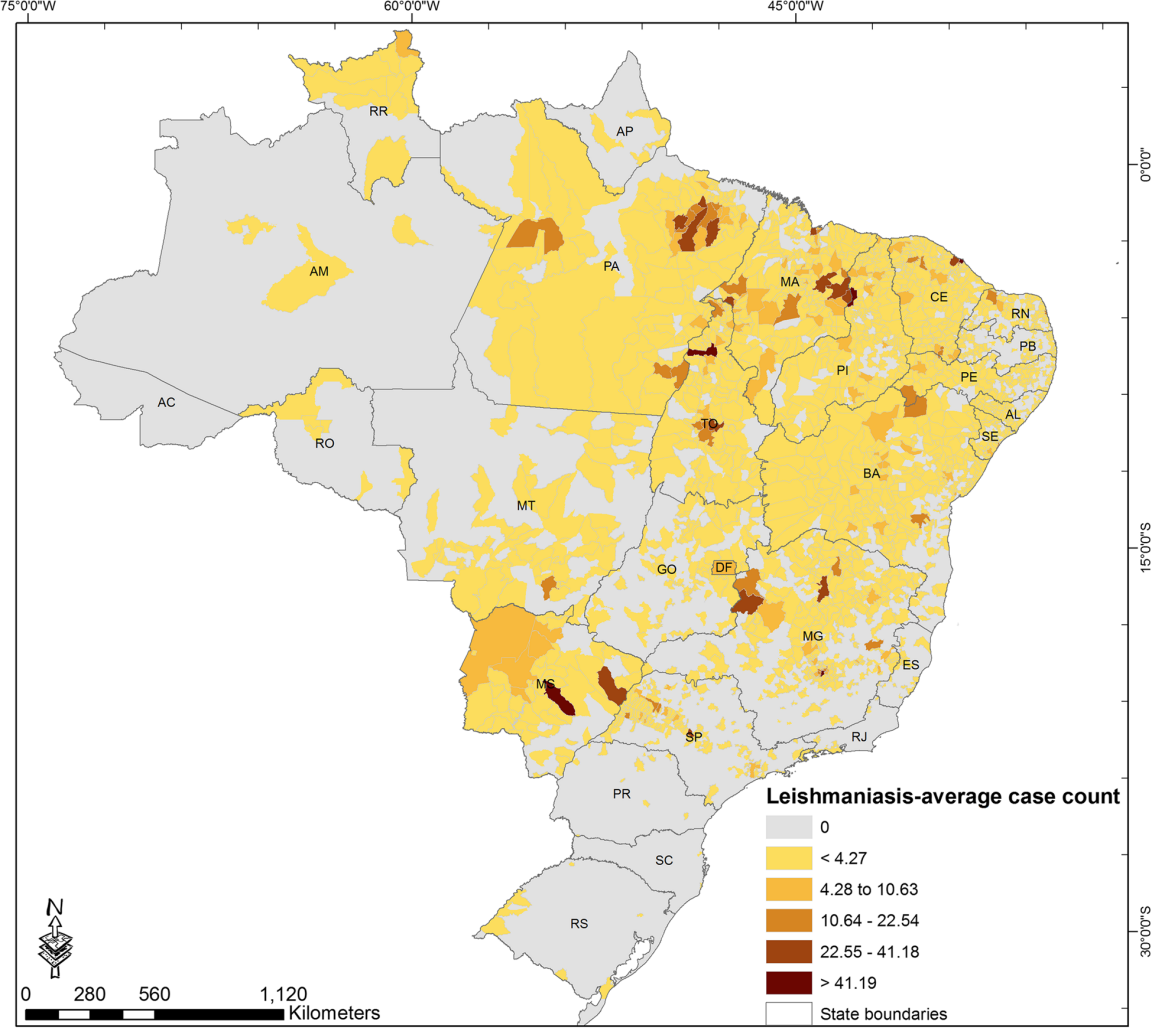
Spatial distribution of the annual average case count of visceral leishmaniasis by municipality, 2004–2014

**Fig. 2:**
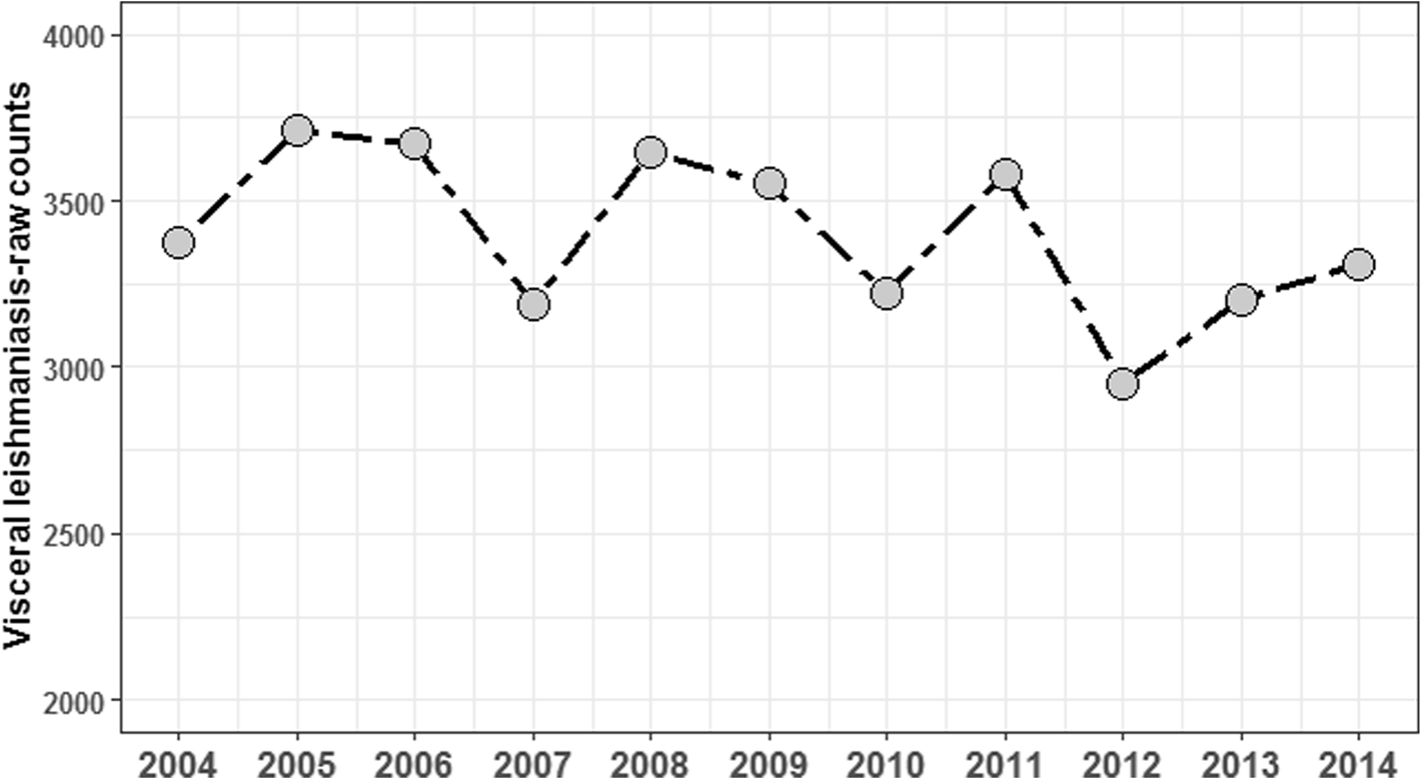
Number of cases of visceral leishmaniasis reported in Brazil over 11 years (2004 to 2014)

From January 2004 to December 2014, a total of 37,405 VL cases were registered by the SINAN/SVS/MH Brazil. The annual average case count by municipality is shown in Fig. 1. The annual case count of VL during the study period (2004–2014), for the entire country, ranged between 2947 and 3713 cases (Fig. 2).

Five municipalities (0.09%) accounted for almost 20% of the total number of cases reported during the period of study: Fortaleza (state of Ceará) 1865 (4.98%), Campo Grande (state of Mato Grosso do Sul) 1520 (4.06%), Araguaína (state of Tocantins) 1294 (3.45%), Belo Horizonte (state of Minas Gerais) 1176 (3.14%), and Teresina (state of Piauí) 961 (2.57%).

### Bayesian hierarchical model

The BHM with Poisson likelihood had the lowest DIC value (Table 1), and included spatial (structured and unstructured), temporal random effects, and interaction term. Models were robust to different choices of priors.

**Table 1:** Composition of eight different models, description of likelihood and for model diagnostics DIC is reported

Model components	Likelihood^a^	DIC (pD)
Log(*θ*_*it*_) = *α* + *υ*_*i*_ + *ν*_*i*_ + *γ*_*t*_	Poisson	59,537.50 (2709.0)
	Zero-inflated negative binomial	74,771.96 (946.49)
	Negative Binomial	75,834.50 (1,8235.2)
	Zero inflated Poisson	78,936.66 (1058.7)
*Log* (*θ*_*it*_) = *α* + *υ*_*i*_ + *ν*_*i*_ + *γ*_*t*_ + *δ*_*it*_	Poisson	49,770.9 (6802.5)
	Zero-inflated negative binomial	75,837.0 (2756.1)
	Negative Binomial	83,372.3 (1,7720.3)
	Zero inflated Poisson	72,654.0 (3166.4)

^a^Additional information about the used likelihood options can be find elsewhere (http://www.r-inla.org/models/latent-models)

**Fig. 3:**
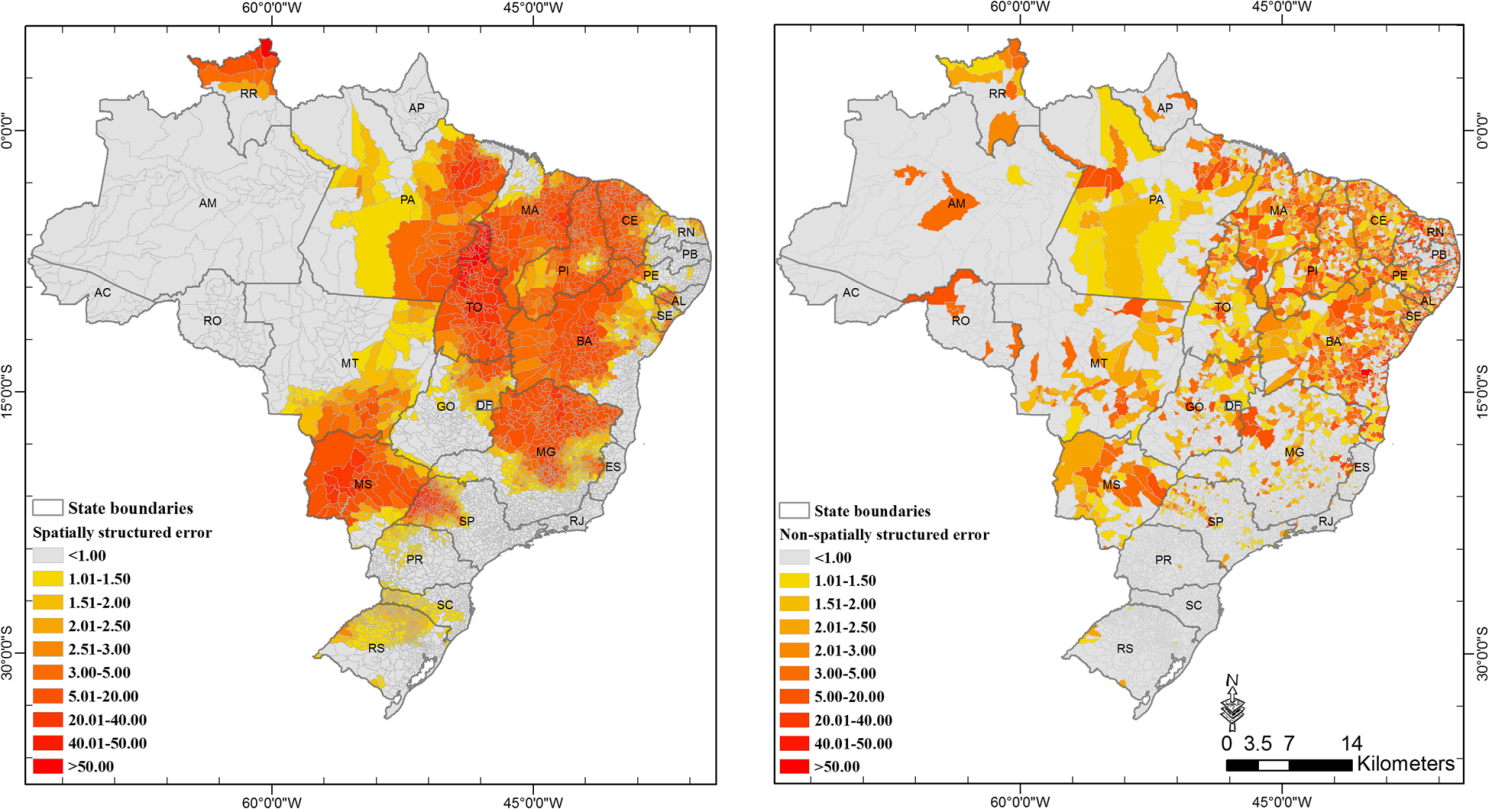
Spatial distribution of the exponentiated spatially structured *υ*_*i*_ (*left*) *and non* − *structured ν*_*i*_ (*right*) *random effects*

The posterior estimates of the spatially structured random effect *u*_*i*_ were higher for municipalities located in the central and eastern part of Brazil, while the non-spatially structured were scattered throughout the country (Fig. 3). The average standard deviation was calculated for all municipalities and *υ*_*i*_ shown to be 2.5 times larger than that of *ν*_*i*_ (6.96 versus 2.76), suggesting that a higher proportion of the unexplained risk of VL (not attributable to the size of the population at risk) was partially explained by factors with a spatial structure (Fig. 3). Finally, the proportion of the marginal variances were calculated for each parameter in the final model: the major contributors were the spatial effects ν (32.8%), υ (57.8%), with less variance explained by the temporal γ (1%) and spatial temporal interaction δ (9.3%).

### Comparisons of the risk classifications

The proportion of municipalities that were classified in the same category by both the BHM via computation of the exceedence probabilities and the SVS/MH classification was 79.84%, very similar to the results obtained when the SVS/MH classification was compared with results using the predicted cases [78.05%, see Additional file 1: Figure S1 and Additional file 2: Figure S2]. This comparison (Table 2) revealed that the classifications based on the BHM (via exceedence probabilities or predicted cases) allocated a higher proportion of municipalities to categories two and three (moderate and intense transmission). Specifically, the classification based on the exceedence probabilities categorized between two and four times more municipalities as category three than the SVS/MH risk classification. Conversely, the current SVS/MH risk classification identified almost four times more municipalities as class one than the classification based on the posterior estimates of the exceedence probabilities. The average agreement between both classifications over the seven years was considered good (weighted Kappa = 0.69) [further information on yearly agreement is provided in Additional file 3: Table S1]. A good agreement (weighted Kappa = 0.63) on average was also obtained when the SVS/MH classification was compared with the one based on the predicted number of cases ($\hat{y_{it}}$) [see Additional file 4: Table S2 for yearly agreement]. However, if the lower risk category (0) was excluded from the comparison the agreement was much lower (0.17 and 0.12 when the SVS/MH classification was compared to the exceedence probabilities and predicted cases from the BHM, respectively), revealing most of the discordant results were obtained in municipalities with some risk as determined by both proposed classification [Table 2 and Additional file 1: Figure S1 to Additional file 2: Figure S2].

**Table 2:** Comparison of the number of municipalities allocated to the different risk levels depending on the classification followed (BHM or SVS/MH classification)

Year	Risk class 0			Risk class 1			Risk class 2			Risk class 3		
	^a^exceedence	^b^SVS/MH	$\hat{y_{it}}$	exceedence	^b^SVS/MH	$\hat{y_{it}}$	exceedence	^b^SVS/MH	$\hat{y_{it}}$	exceedence	^b^SVS/MH	$\hat{y_{it}}$
2008	4691	4307	5032	244	1040	16	233	88	242	396	129	272
2009	4659	4329	5033	259	1006	17	243	99	227	403	130	285
2010	4626	4312	5012	266	1016	21	252	98	230	420	138	299
2011	4614	4289	5003	266	1003	15	259	129	230	425	143	309
2012	4619	4259	4994	265	1060	22	252	146	240	426	99	301
2013	4604	4275	4985	263	1034	21	234	105	232	463	150	321
2014	4527	4273	4949	272	1026	12	271	119	247	494	146	354

^a^Number of municipalities classified as 0, 1, 2, and 3 based on the posterior estimates of exceedence probabilities (**0:** < 0.5, **1:** 0.5–0.75, **2:** 0.75–0.95 and **3:** > 0.95). ^b^Number of municipalities classified as 0- zero case reported; 1-sliding average lower than 2.4; 2-sliding average was between [2.4 to 4.4); and 3– sliding average above or equal to 4.4 cases by the MH according to the current national regulations

**Fig. 4:**
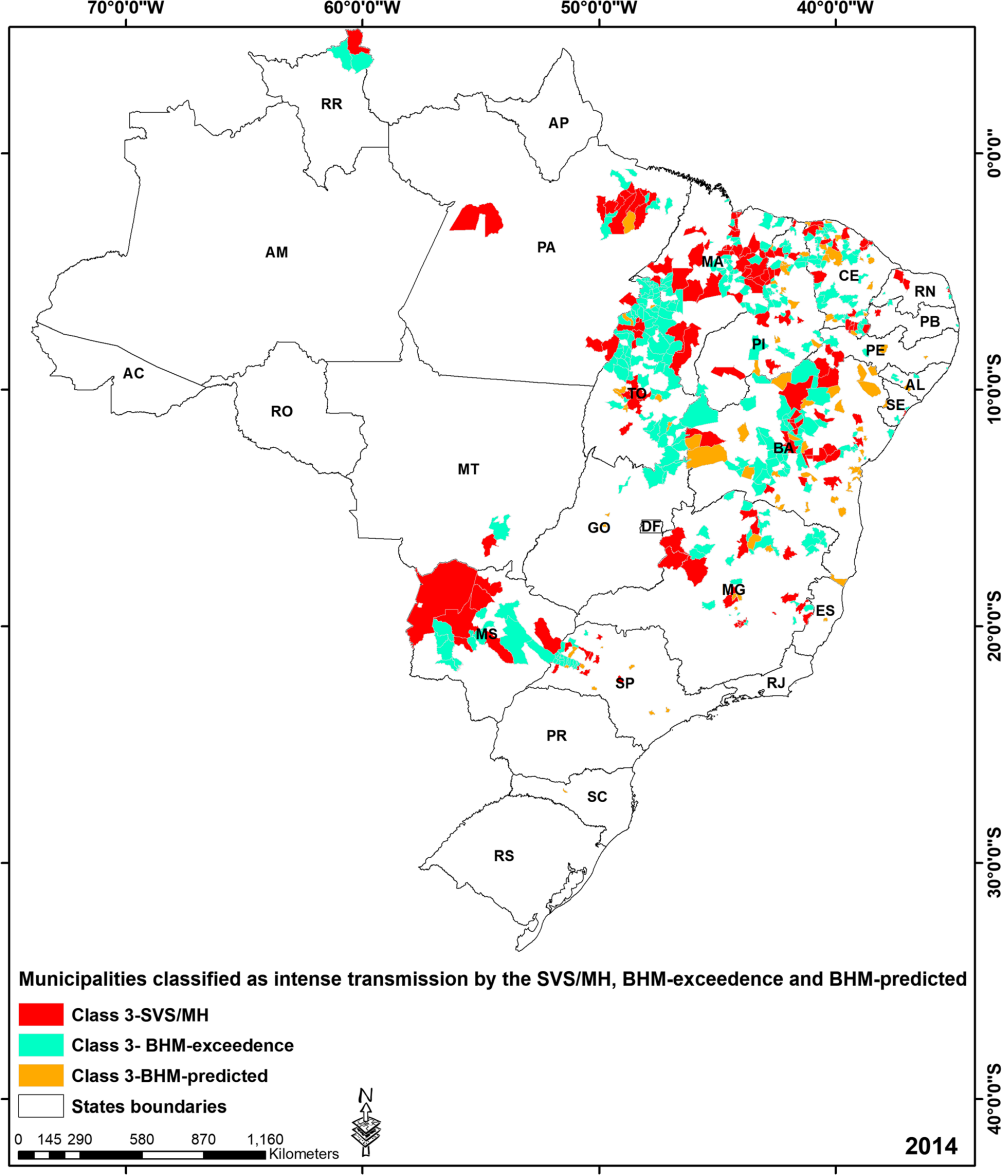
Geographic patterns of the municipalities classified as high-risk (class 3) by the SVS/MH, BHM- exceedence and BHM-predicted predicted number of cases ($\hat{y_{it}}$) by the BHM

We have explored the spatial distribution for the comparison among all classifications, we demonstrate the scenario for the 2014 pattern, where SVS/MH, BHM-exceedence and BHM-predictions for intense transmission (class 3) are mapped in Fig. 4 [see Additional file 5: Figure S3, Additional file 6: Figure S4, Additional file 7: Figure S5, Additional file 8: Figure S6, Additional file 9: Figure S7, Additional file 10: Figure S8 for the 2008 to 2013 maps], showing that discordant municipalities were located throughout the country.

## Discussion

VL is endemic in Brazil, and has been historically distributed across multiple states, especially in the North and Northeast regions of the country. However, recent reports indicate that the disease is expanding within Brazil and is reaching neighboring countries like Argentina and Uruguay [23–25]. Recently affected areas in Brazil include states located in the South (such as Rio Grande do Sul) and in the Midwest region [10]. For the study period, municipalities that presented higher number of cases were mostly located in the states of Tocantins, Minas Gerais, Mato Grosso do Sul, Ceará and Piauí (Fig. 1), supporting the results observed in previous studies that had also identified the above states as high-risk areas [26–30]. For the 11 years studied here less than 10% of the municipalities reported at least one case of VL in any given year (mean of municipalities with one or more VL cases during 2004–2014 = 437, min = 380, max = 492). However, VL incidence varied largely in those affected municipalities.

The inclusion of both spatially structured and unstructured random effects in the model allowed a better understanding of how the risk was directly explained by the population at risk across the country. The exponentiated posterior estimates for the spatially structured random effect term were above one in multiple regions including Central-Western, Northeast and especially north of Roraima state (Fig. 3-left). High values of *u*_*i*_ indicate a positive association between the spatially structured effects and VL in Brazil, signaling the presence of additional risk factors that are not directly related with VL occurrence and that have a spatial component. This spatially-dependent risk may be in part related with the local density of infected reservoirs (dogs), in line with previous studies that described a positive spatial dependency between the occurrence of human and canine VL cases [31]. Therefore, larger concentrations of infected dogs per inhabitants in certain municipalities could lead to increased risk, since dogs are considered the main reservoir of the disease in Latin America and in Brazil in particular [27, 32, 33].

Increased risk may be also explained by other factors. For example, in some areas with high VL incidence like Teresina (Northeastern Brazil) a correlation between VL incidence and more limited urban infrastructures and poorer living conditions has been previously described [26, 34, 35]. Future analysis can expand on our models by incorporating covariates explaining local development as one example. Changes in the environment, such as deforestation due to expansion of the road networks, have been also shown to have a major effect on the risk of VL and other vector-borne diseases [36]. Indeed, the expanding habitat of the vector may be associated to some extent with the increase in VL incidence in areas traditionally considered non-endemic in Brazil, especially in the South and Midwest regions, a situation that may become more concerning in the future [25].

The nearly 80% agreement between the SVS/MH and BHM-exceedence and predicted risk classifications when all risk categories are considered suggests that the current strategy for the classification of municipalities may provide an acceptable approach in a significant proportion of the municipalities in the country. However, when results from municipalities classified in categories 1–3 (i.e., ‘some risk’) by the three approaches were compared, the agreement dropped largely [Table 2, Additional file 1: Figure S1 and Additional file 2: Figure S2], and major disagreements were identified particularly regarding to the category of higher risk (class 3) as classified by the BHM, that were evident throughout the study period [Additional file 3: Table S1, Additional file 4: Table S2, Fig. 4 and Additional file 5: Figure S3, Additional file 6: Figure S4, Additional file 7: Figure S5, Additional file 8: Figure S6, Additional file 9: Figure S7, Additional file 10: Figure S8 for the 2008 to 2013 maps]: a considerable proportion of these high risk municipalities (between 58% in 2012 and 82% in 2013) were identified to have lower risk according to the SVS/MH classification. The SVS/MH classification seemed to be more sensitive to year-to-year changes (for example, there was a 30% drop in the number of municipalities classified as high risk between 2011 and 2012), which could be due to surveillance artifacts since the risk of VL would not be expected to change so drastically in such a short time-span. The classification yielded by the BHM, on the other hand, provided a more stable risk landscape over time and space due to the smoothing stemming from the inclusion of spatial effects in the model [Fig. 4 and Additional file 5: Figure S3, Additional file 6: Figure S4, Additional file 7: Figure S5, Additional file 8: Figure S6, Additional file 9: Figure S7, Additional file 10: Figure S8]. This is obvious from a close look at the municipalities classified differently by the two approaches, showing that these were typically located neighboring others with a large spatially structured random effect term (*υ*_*i*_).The implications in the control of VL may be relevant if municipalities stop the application of control measures without accounting for the risk in neighboring municipalities (Fig. 4).

Both “moderate” and “intense transmission” municipalities according to SVS/MH (categories 2 and 3) are subjected to the same disease control measures in terms of resources and active surveillance activities. However, the BHM results suggest that a substantial underestimation may take place when only focusing on numerator data, since every year an average of 131 and 288 additional municipalities were classified as moderate (class 2) and intense (class 3) transmission areas, respectively, using this approach. This highlights the importance of incorporating information on the population at risk as well as spatial and temporal effects most related to the risk of infectious diseases. The comparison between the SVS/MH classification and those based on the exceedence probabilities or the predicted number of cases ($\hat{y_{it}}$) revealed that even though agreement was good (weighted Kappa min:0.66-max:0.69) discordances were not only found in municipalities classified as higher risk [Additional file 3: Table S1, Additional file 4: Table S2]. Our current analyses allow the identification of municipalities with higher VL risk that could have been previously inadequately classified according to the methodology adopted by the SVS/MH. The new classification proposed in this study may help to identify municipalities that, despite not presenting high morbidity, are under a high risk of disease transmission, and should therefore be subjected to improved surveillance.

Finally, the limitations of this study are mainly associated to the lack of information on neighboring countries for municipalities located at the edge of the study area (Paraguay, Argentina and Bolivia). In addition, location of cases were based on where the notification took place, and may not indicate where the infection actually occurred. However, we suggest that the modeling the incidence ratio and inclusion of spatial and temporal effects and the smoothing technique we used helped to remove the effects of the variation of count cases used by the current MHS risk classification, and hence provide a better approximation of the municipality-level risk.

## Conclusions

The comparison between the VL risk classification currently in use by the SVS/MH and that obtained through a BHM revealed that raw case counts of VL may be sufficient to indicate disease risk in a large proportion of the municipalities in Brazil, but may underestimate the risk in others, particularly those neighboring high risk areas. Our results identified “hot” areas where disease clustered, and where control and surveillance efforts could be implemented in order to prevent further spread of VL in the country. Resources to support increased measures in those hot areas could come from the many more areas classified as “1” (sporadic transmission) by the SVS/MH compared to those identify by our models.

## Additional files

Additional file 1: **Figure S1.** Proportion of municipalities classified by the BHM model exceedence probabilities and the SVS/MH classification. (TIFF 26367 kb)

Additional file 2: **Figure S2.** Proportion of municipalities classified by the BHM model-predicted risk class and the SVS/MH classification. (TIFF 26367 kb)

Additional file 3: **Table S1.** Weighted Kappa between BHM model-exceedence probabilities and the SVS/MH classification. (DOCX 18 kb)

Additional file 4: **Table S2.** Weighted Kappa between BHM model-predicted risk class and the SVS/MH classification. (DOCX 18 kb)

Additional file 5: **Figure S3.** The spatial distribution of all classifications SVS/MH, BHM-exceedence and BHM-predictions for 2008. (TIF 26986 kb)

Additional file 6: **Figure S4.** The spatial distribution of all classifications SVS/MH, BHM-exceedence and BHM-predictions for 2009. (TIF 26986 kb)

Additional file 7: **Figure S5.** The spatial distribution of all classifications SVS/MH, BHM-exceedence and BHM-predictions for 2010. (TIF 26986 kb)

Additional file 8: **Figure S6.** The spatial distribution of all classifications SVS/MH, BHM-exceedence and BHM-predictions for 2011. (TIF 26986 kb)

Additional file 9: **Figure S7.** The spatial distribution of all classifications SVS/MH, BHM-exceedence and BHM-predictions for 2012. (TIF 26986 kb)

Additional file 10: **Figure S8.** The spatial distribution of all classifications SVS/MH, BHM-exceedence and BHM-predictions for 2013. (TIF 26986 kb)

## Abbreviations

BHM: Bayesian hierarchical model
DIC: Deviance Information Criterion
IBGE: Instituto Brasileiro de Geografia e Estatística
INLA: Integrated Nested Laplace Approximation
RW1: Random walk type 1
SIR: Standardized incidence ratios
SVS/MH: Secretaria de Vigilância em Saúde of Brazil’s Ministry of Health
VL: Visceral leishmaniasis
