# Supplementary figures and images for: Revisiting area risk classification of visceral leishmaniasis in Brazil
Source: BMC Infect Dis. 2019 Jan 3;19:2. doi: 10.1186/s12879-018-3564-0 (PMC6318941; doi:10.1186/s12879-018-3564-0)

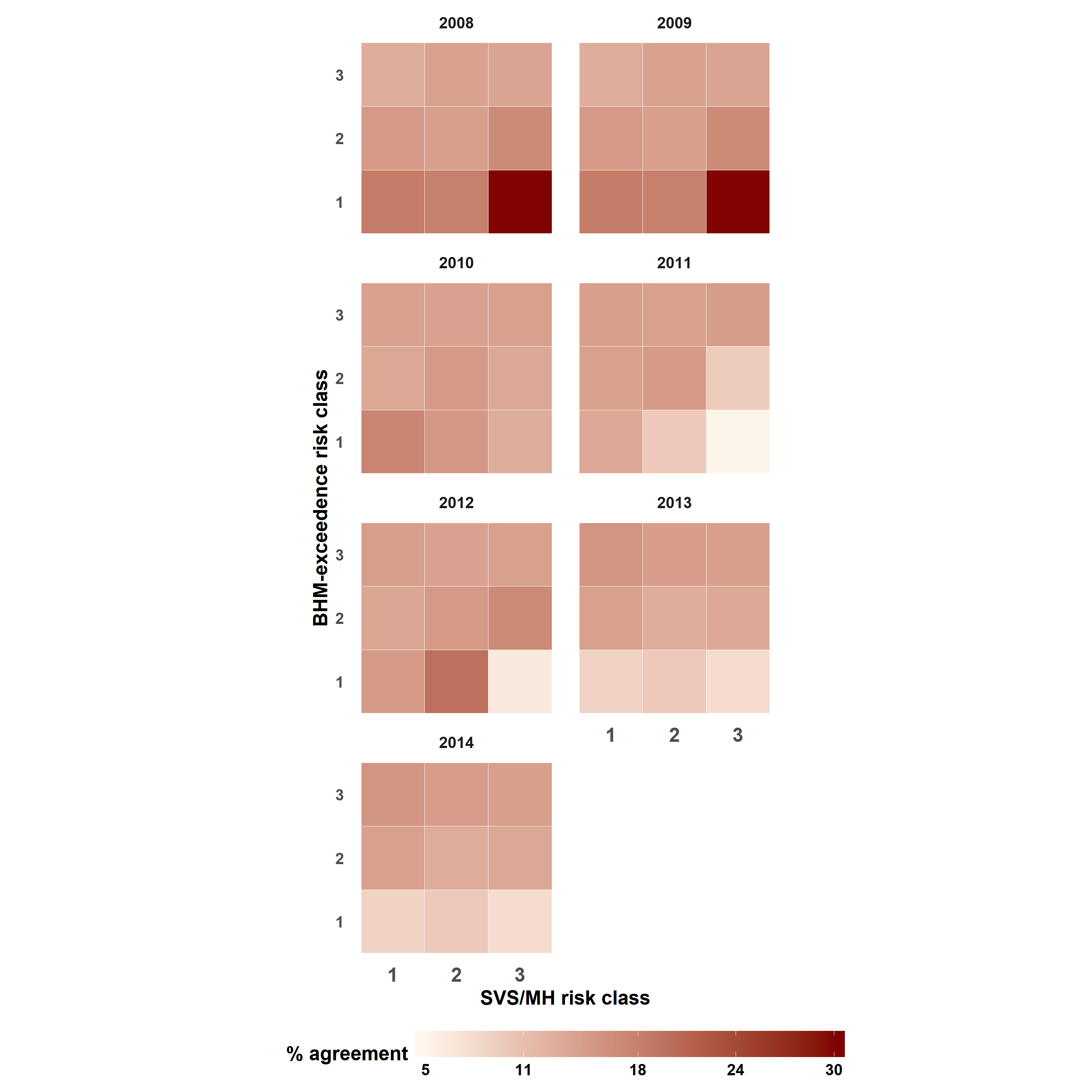

Supplement: Supplementary file 1 — Figure S1. Proportion of municipalities classified by the BHM model exceedence probabilities and the SVS/MH classification. (TIFF 26367 kb) [file 12879_2018_3564_MOESM1_ESM.tiff]

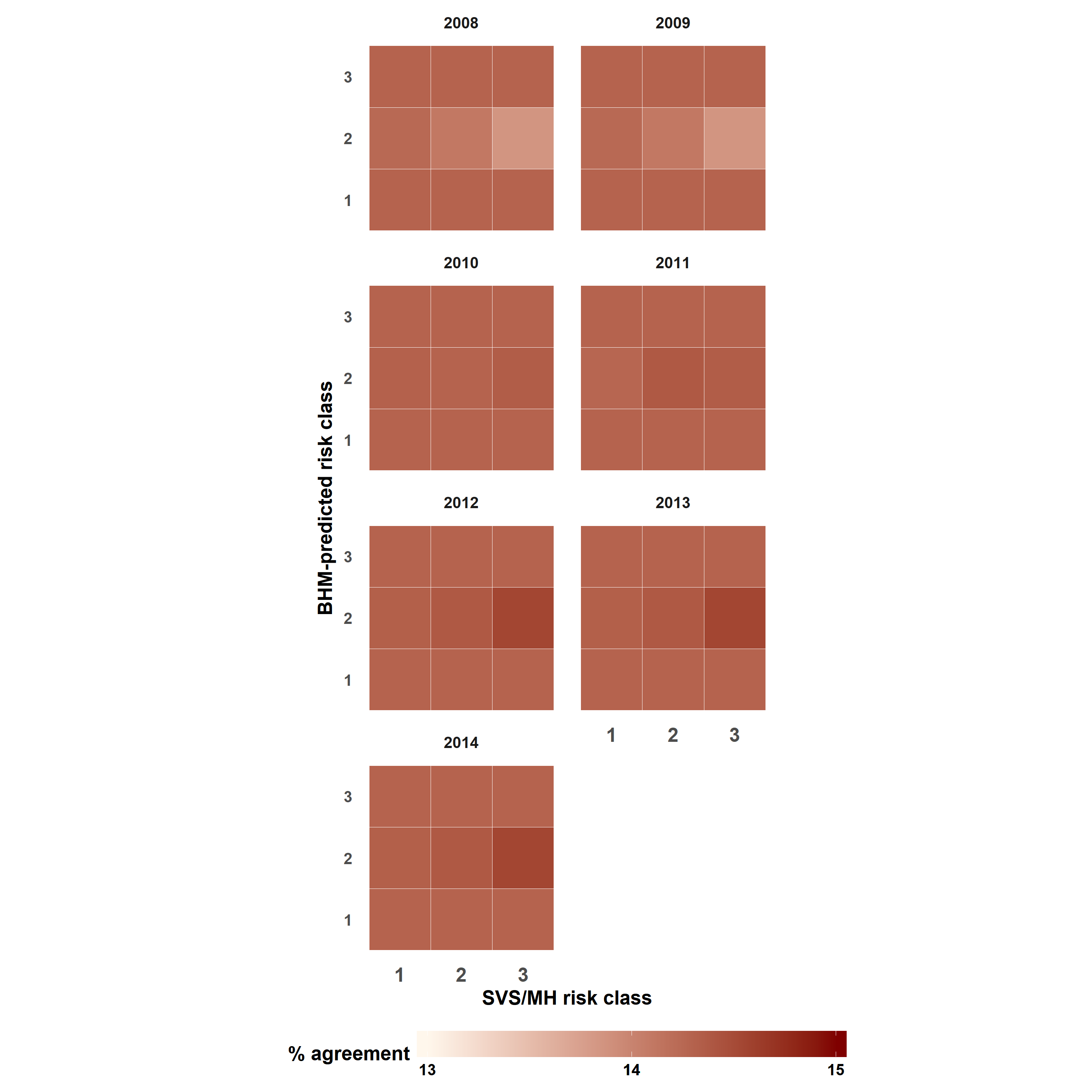

Supplement: Supplementary file 2 — Figure S2. Proportion of municipalities classified by the BHM model-predicted risk class and the SVS/MH classification. (TIFF 26367 kb) [file 12879_2018_3564_MOESM2_ESM.tiff]

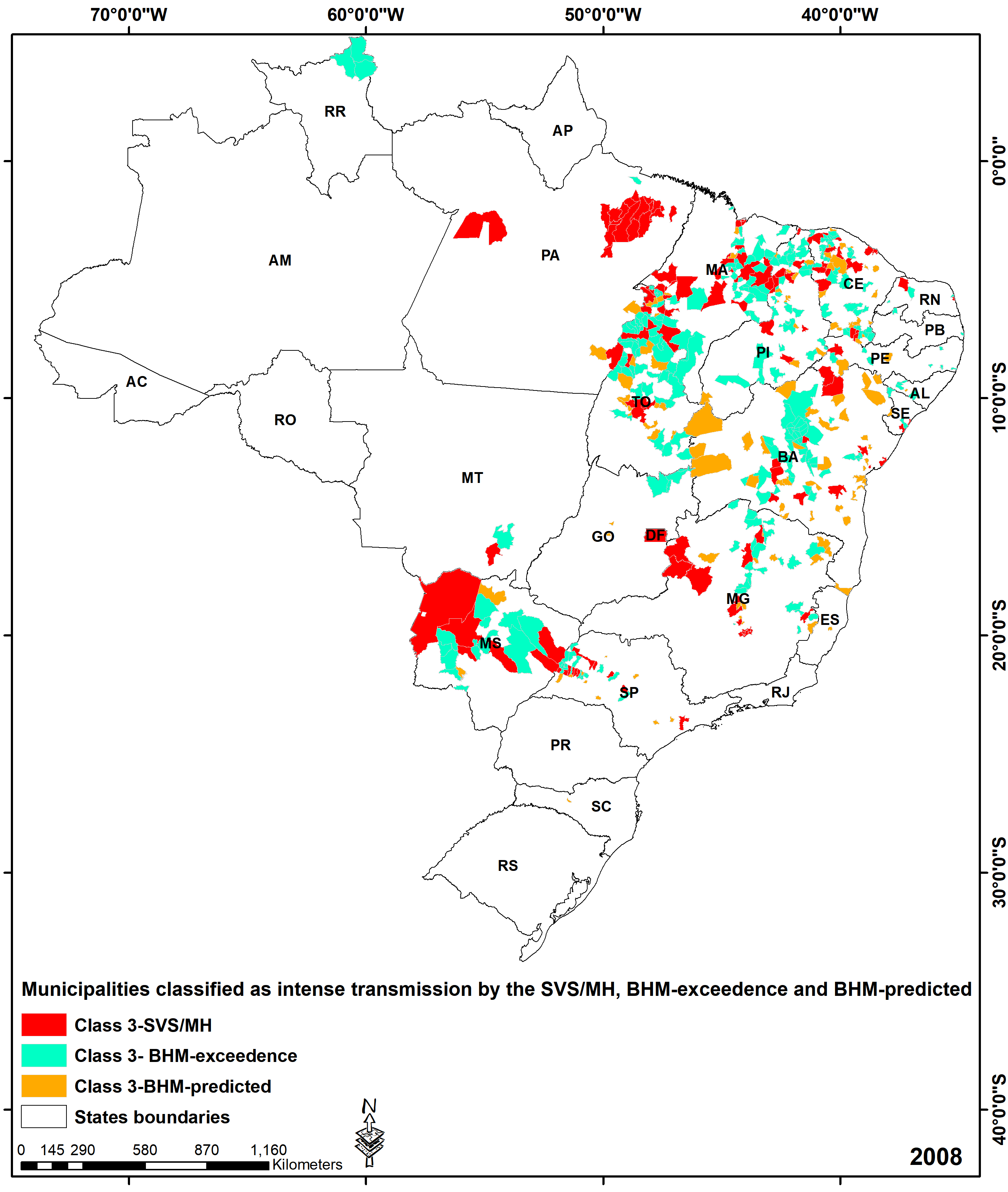

Supplement: Supplementary file 5 — Figure S3. The spatial distribution of all classifications SVS/MH, BHM-exceedence and BHM-predictions for 2008. (TIF 26986 kb) [file 12879_2018_3564_MOESM5_ESM.tif]

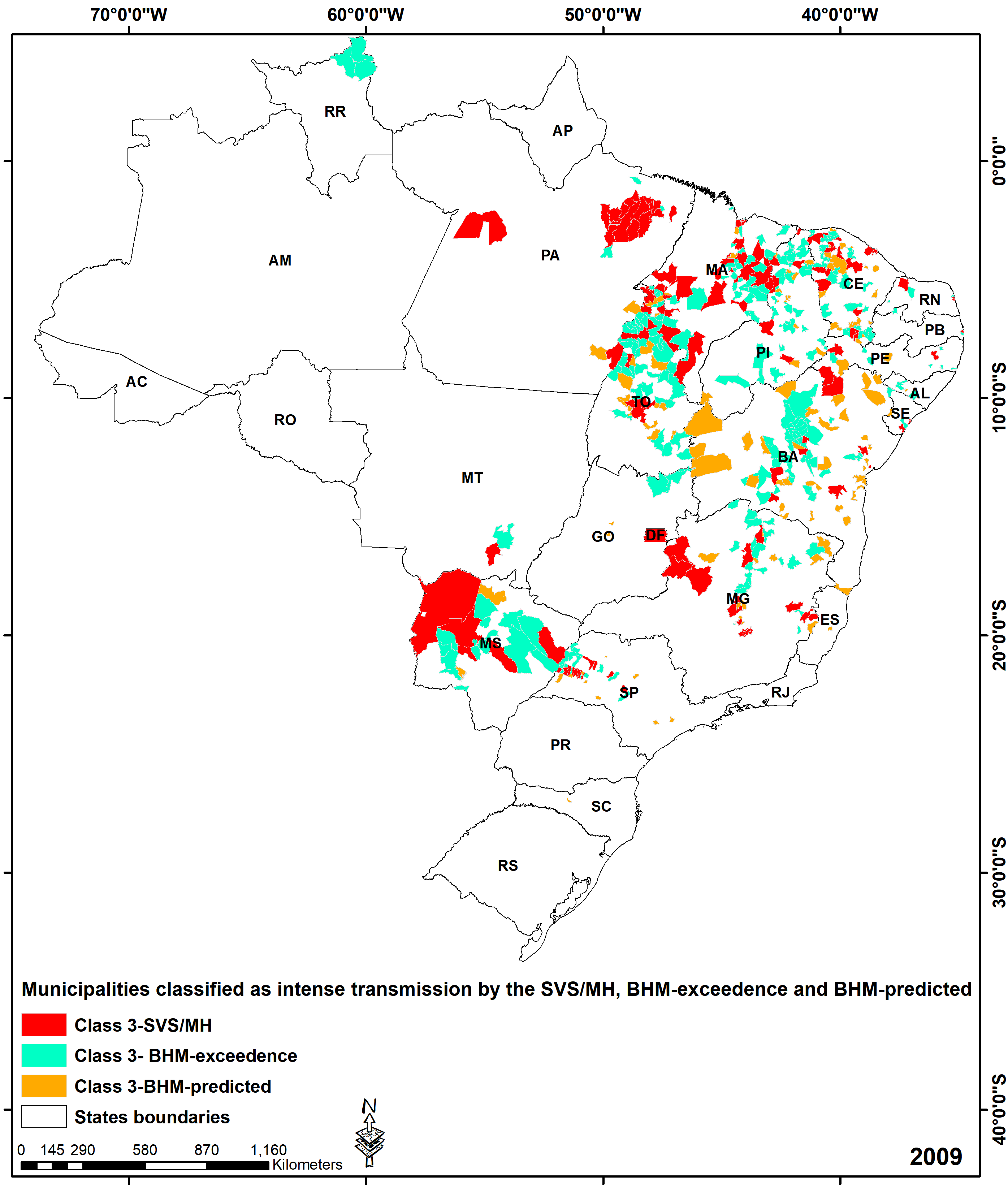

Supplement: Supplementary file 6 — Figure S4. The spatial distribution of all classifications SVS/MH, BHM-exceedence and BHM-predictions for 2009. (TIF 26986 kb) [file 12879_2018_3564_MOESM6_ESM.tif]

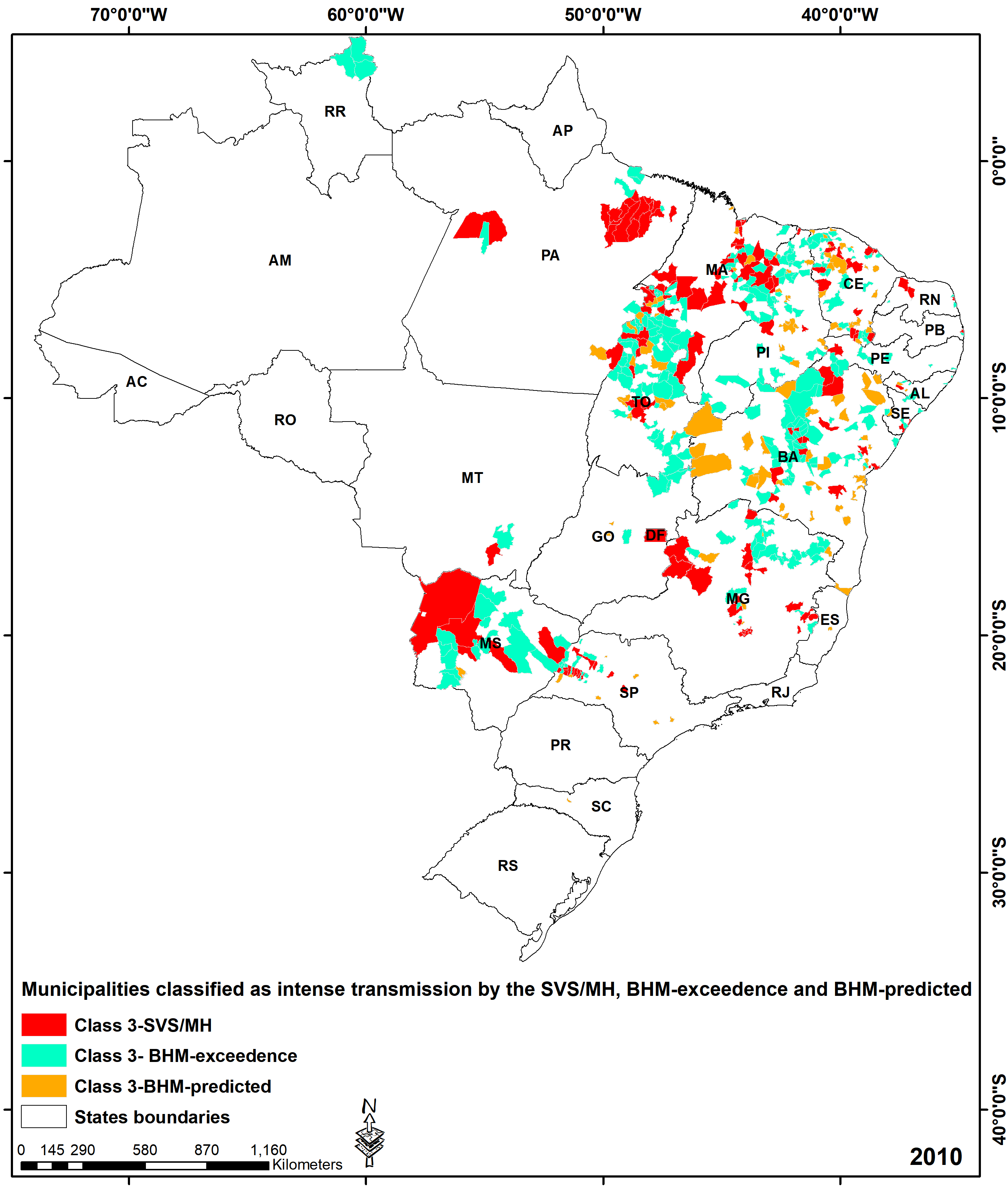

Supplement: Supplementary file 7 — Figure S5. The spatial distribution of all classifications SVS/MH, BHM-exceedence and BHM-predictions for 2010. (TIF 26986 kb) [file 12879_2018_3564_MOESM7_ESM.tif]

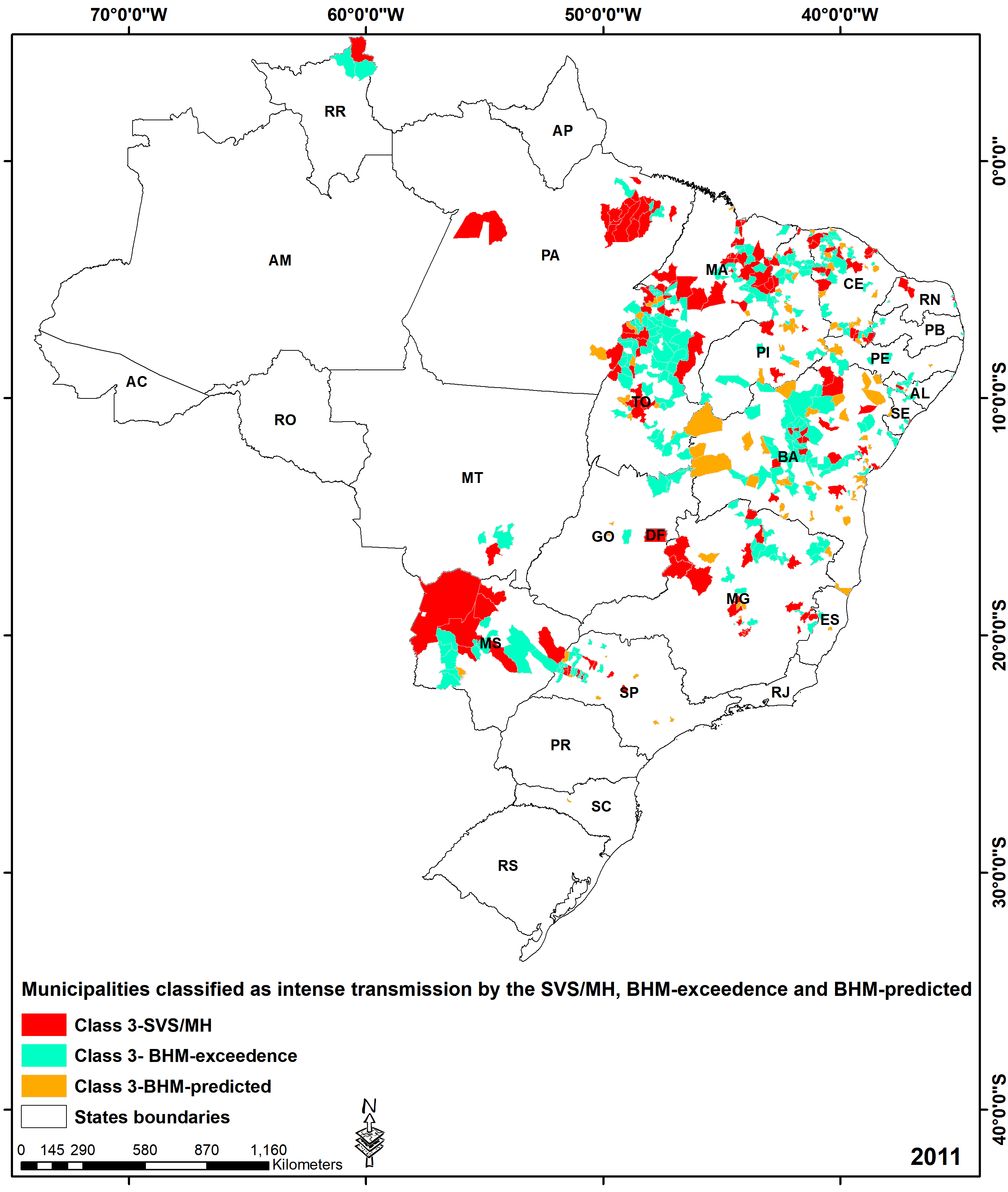

Supplement: Supplementary file 8 — Figure S6. The spatial distribution of all classifications SVS/MH, BHM-exceedence and BHM-predictions for 2011. (TIF 26986 kb) [file 12879_2018_3564_MOESM8_ESM.tif]

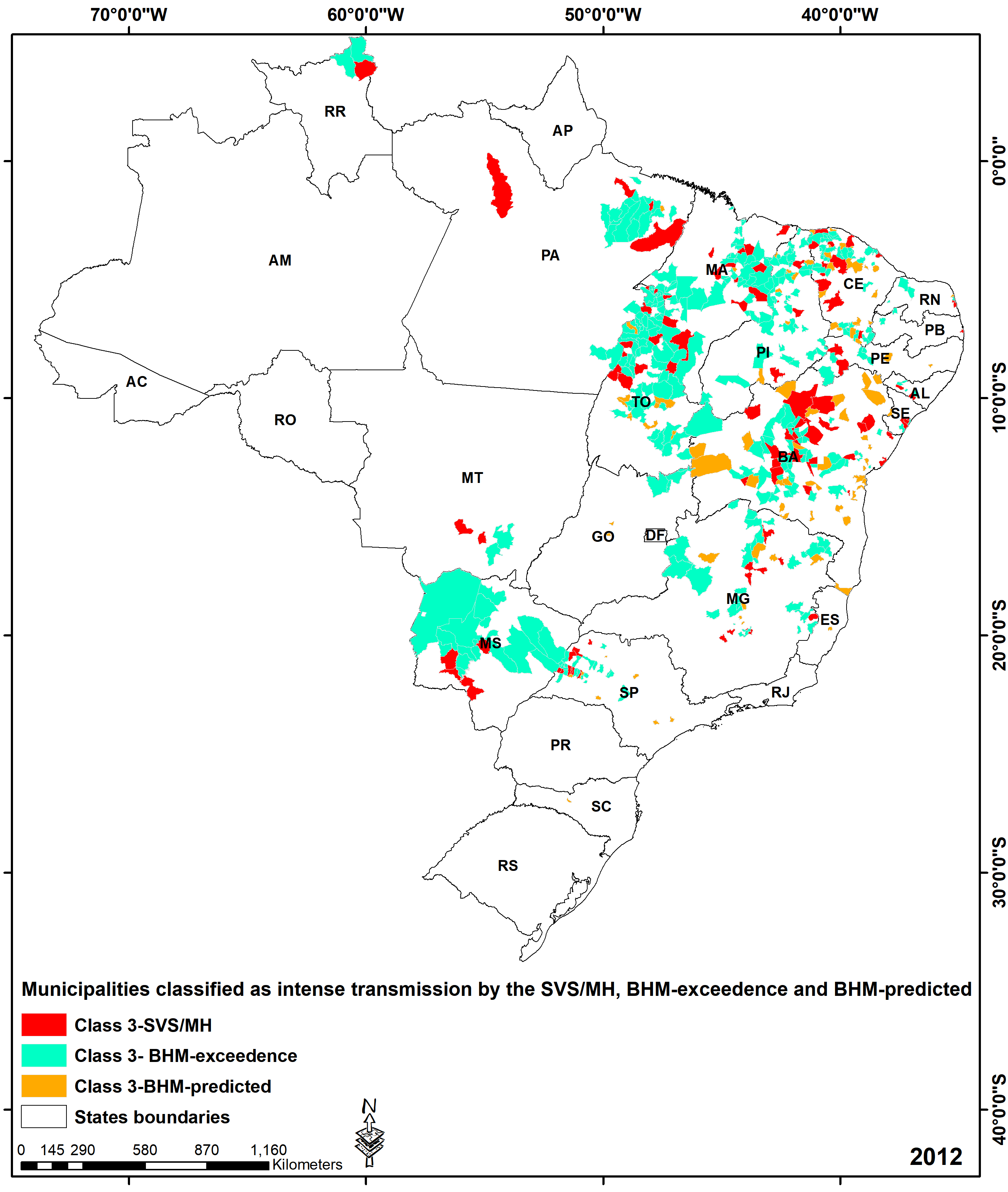

Supplement: Supplementary file 9 — Figure S7. The spatial distribution of all classifications SVS/MH, BHM-exceedence and BHM-predictions for 2012. (TIF 26986 kb) [file 12879_2018_3564_MOESM9_ESM.tif]

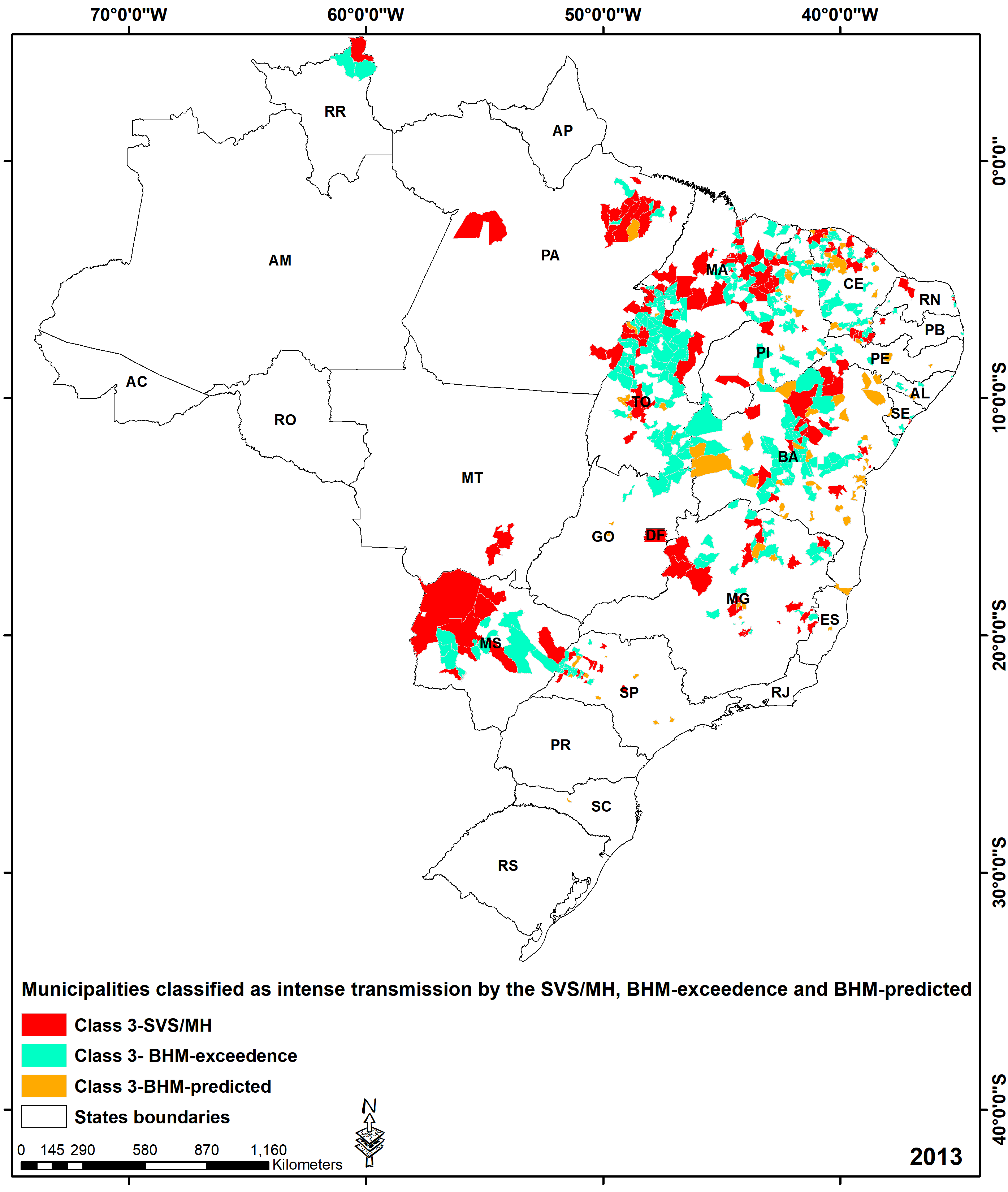

Supplement: Supplementary file 10 — Figure S8. The spatial distribution of all classifications SVS/MH, BHM-exceedence and BHM-predictions for 2013. (TIF 26986 kb) [file 12879_2018_3564_MOESM10_ESM.tif]
