# Supplementary material for: Revisiting area risk classification of visceral leishmaniasis in Brazil
Source: BMC Infect Dis. 2019 Jan 3;19:2. doi: 10.1186/s12879-018-3564-0 (PMC6318941; doi:10.1186/s12879-018-3564-0)
Supplement: Supplementary file 3 — Table S1. Weighted Kappa between BHM model-exceedence probabilities and the SVS/MH classification. (DOCX 18 kb) [file 12879_2018_3564_MOESM3_ESM.docx]

| **Exceedence** | **SVS/MH -2008 to 2014** | | | | Total |
| --- | --- | --- | --- | --- | --- |
|  | **0** | **1** | **2** | **3** |  |
| 0 | **202526** | 24410 | 456 | 950 | 227696 |
| 1 | 4530 | **7832** | 271 | 9552 | 12782 |
| 2 | 2302 | 8501 | **656** | 4105 | 11837 |
| 3 | 950 | 9552 | 378 | **5714** | 20321 |
| Total | 210308 | 50295 | 5488 | 6545 | **272693** |
| Weighted Kappa = 0.69 | | | | | |
| **Exceedence** | **SVS/MH -2008** | | | | Total |
|  | **0** | **1** | **2** | **3** |  |
| 0 | **4142** | 536 | 7 | 6 | 4691 |
| 1 | 94 | **142** | 6 | 2 | 244 |
| 2 | 53 | 163 | **13** | 4 | 233 |
| 3 | 18 | 199 | 62 | **117** | 396 |
| Total | 4307 | 1040 | 88 | 129 | **5564** |
| Weighted Kappa = 0.66 | | | | | |
| **Exceedence** | **SVS/MH -2009** | | | | Total |
|  | **0** | **1** | **2** | **3** |  |
| 0 | **4168** | 478 | 7 | 6 | 4659 |
| 1 | 91 | **159** | 4 | 5 | 259 |
| 2 | 57 | 171 | **11** | 4 | 243 |
| 3 | 13 | 198 | 77 | **115** | 403 |
| Total | 4329 | 1006 | 99 | 130 | **5564** |
| Weighted Kappa = 0.67 | | | | | |
| **Exceedence** | **SVS/MH -2010** | | | | Total |
|  | **0** | **1** | **2** | **3** |  |
| 0 | **4179** | 438 | 5 | 4 | 4626 |
| 1 | 85 | **176** | 2 | 3 | 266 |
| 2 | 38 | 195 | **15** | 4 | 252 |
| 3 | 10 | 207 | 76 | **127** | 420 |
| Total | 4312 | 1016 | 98 | 138 | **5564** |
| Weighted Kappa = 0.70 | | | | | |
| **Exceedence** | **SVS/MH -2011** | | | | Total |
|  | **0** | **1** | **2** | **3** |  |
| 0 | **4142** | 464 | 5 | 3 | 4614 |
| 1 | 90 | **166** | 10 | 0 | 266 |
| 2 | 48 | 190 | **15** | 6 | 259 |
| 3 | 9 | 183 | 99 | **134** | 425 |
| Total | 4289 | 1003 | 129 | 143 | **5564** |
| Weighted Kappa = 0.71 | | | | | |
| **Exceedence** | **SVS/MH -2012** | | | | Total |
|  | **0** | **1** | **2** | **3** |  |
| 0 | **4124** | 486 | 3 | 6 | 4619 |
| 1 | 87 | **173** | 2 | 5 | 265 |
| 2 | 38 | 197 | **7** | 10 | 252 |
| 3 | 10 | 204 | 134 | **78** | 426 |
| Total | 4259 | 1060 | 146 | 99 | **5564** |
| Weighted Kappa = 0.68 | | | | | |
| **Exceedence** | **SVS/MH -2013** | | | | Total |
|  | **0** | **1** | **2** | **3** |  |
| 0 | **4134** | 455 | 10 | 5 | 4604 |
| 1 | 88 | **172** | 3 | 0 | 263 |
| 2 | 42 | 177 | **12** | 3 | 234 |
| 3 | 11 | 230 | 80 | **142** | 463 |
| Total | 4275 | 1034 | 105 | 150 | **5564** |
| Weighted Kappa = 0.70 | | | | | |
| **Exceedence** | **SVS/MH -2014** | | | | Total |
|  | **0** | **1** | **2** | **3** |  |
| 0 | **4116** | 401 | 7 | 3 | 4527 |
| 1 | 102 | **169** | 1 | 0 | 272 |
| 2 | 47 | 211 | **10** | 3 | 271 |
| 3 | 8 | 245 | 101 | **140** | 494 |
| Total | 4273 | 1026 | 119 | 146 | **5564** |
| Weighted Kappa = 0.71 | | | | | |
