# Supplementary material for: Revisiting area risk classification of visceral leishmaniasis in Brazil
Source: BMC Infect Dis. 2019 Jan 3;19:2. doi: 10.1186/s12879-018-3564-0 (PMC6318941; doi:10.1186/s12879-018-3564-0)
Supplement: Supplementary file 4 — Table S2. Weighted Kappa between BHM model-predicted risk class and the SVS/MH classification. (DOCX 18 kb) [file 12879_2018_3564_MOESM4_ESM.docx]

| **BHM (predicted cases)** | **SVS/MH -2008-2014** | | | | Total |
| --- | --- | --- | --- | --- | --- |
|  | **0** | **1** | **2** | **3** |  |
| **0** | **1443736** | 227017 | 6223 | 4410 | 1681386 |
| **1** | 2044 | **4067** | 455 | 98 | 6664 |
| **2** | 17360 | 59731 | **7609** | 7175 | 91875 |
| **3** | 9016 | 61103 | 24031 | **34034** | 128184 |
| **Total** | 1472156 | 351918 | 38318 | 45717 | 1908109 |
| Weighted Kappa = 0.63 | | | | | |
| **BHM (predicted cases)** | **SVS/MH -2008** | | | | Total |
|  | **0** | **1** | **2** | **3** |  |
| **0** | **4256** | 748 | 16 | 12 | 5032 |
| **1** | 3 | **12** | 1 | 0 | 16 |
| **2** | 36 | 160 | **31** | 15 | 242 |
| **3** | 12 | 120 | 40 | **100** | 272 |
| **Total** | 4307 | 1040 | 88 | 127 | 5562 |
| Weighted Kappa = 0.62 | | | | | |
| **BHM (predicted cases)** | **SVS/MH -2009** | | | | Total |
|  | **0** | **1** | **2** | **3** |  |
| **0** | **4285** | 720 | 15 | 13 | 5033 |
| **1** | 3 | **12** | 2 | 0 | 17 |
| **2** | 32 | 153 | **27** | 15 | 227 |
| **3** | 9 | 121 | 55 | **100** | 285 |
| **Total** | 4329 | 1006 | 99 | 128 | 5562 |
| Weighted Kappa = 0.66 | | | | | |
| **BHM (predicted cases)** | **SVS/MH -2010** | | | | Total |
|  | **0** | **1** | **2** | **3** |  |
| **0** | **4282** | 706 | 12 | 12 | 5012 |
| **1** | 0 | **21** | 0 | 0 | 21 |
| **2** | 22 | 164 | **24** | 20 | 230 |
| **3** | 8 | 125 | 62 | **104** | 299 |
| **Total** | 4312 | 1016 | 98 | 136 | 5562 |
| Weighted Kappa = 0.68 | | | | | |
| **BHM (predicted cases)** | **SVS/MH -2011** | | | | Total |
|  | **0** | **1** | **2** | **3** |  |
| **0** | **4254** | 719 | 19 | 11 | 5003 |
| **1** | 4 | **8** | 3 | 0 | 15 |
| **2** | 25 | 149 | **31** | 25 | 230 |
| **3** | 6 | 127 | 74 | **102** | 309 |
| **Total** | 4289 | 1003 | 127 | 138 | 5557 |
| Weighted Kappa = 0.68 | | | | | |
| **BHM (predicted cases)** | **SVS/MH -2012** | | | | Total |
|  | **0** | **1** | **2** | **3** |  |
| **0** | **4230** | 740 | 11 | 13 | 4994 |
| **1** | 3 | **12** | 3 | 4 | 22 |
| **2** | 18 | 178 | **21** | 23 | 240 |
| **3** | 8 | 130 | 105 | **58** | 301 |
| **Total** | 4259 | 1060 | 140 | 98 | 5557 |
| Weighted Kappa = 0.65 | | | | | |
| **BHM (predicted cases)** | **SVS/MH -2013** | | | | Total |
|  | **0** | **1** | **2** | **3** |  |
| **0** | **4239** | 710 | 21 | 15 | 4985 |
| **1** | 5 | **16** | 0 | 0 | 21 |
| **2** | 23 | 162 | **24** | 23 | 232 |
| **3** | 8 | 146 | 60 | **107** | 321 |
| **Total** | 4275 | 1034 | 105 | 145 | 5559 |
| Weighted Kappa = 0.66 | | | | | |
| **BHM (predicted cases)** | **SVS/MH -2014** | | | | Total |
|  | **0** | **1** | **2** | **3** |  |
| **0** | **4244** | 682 | 13 | 10 | 4949 |
| **1** | 0 | **11** | 0 | 1 | 12 |
| **2** | 24 | 174 | **24** | 25 | 247 |
| **3** | 5 | 159 | 81 | **109** | 354 |
| **Total** | 4273 | 1026 | 118 | 145 | 5562 |
| Weighted Kappa = 0.69 | | | | | |
